# Exploring Proton-Only NMR Experiments and Filters for Daphnia In Vivo: Potential and Limitations

**DOI:** 10.3390/molecules28124863

**Published:** 2023-06-20

**Authors:** Kiera Ronda, Katelyn Downey, Amy Jenne, Monica Bastawrous, William W. Wolff, Katrina Steiner, Daniel H. Lysak, Peter M. Costa, Myrna J. Simpson, Karl J. Jobst, Andre J. Simpson

**Affiliations:** 1 Department of Physical & Environmental Sciences, University of Toronto Scarborough, 1265 Military Trail, Toronto, ON M1C 1A4, Canada; 2 Department of Chemistry, Memorial University of Newfoundland, 45 Arctic Ave., St. John’s, NL A1C 5S7, Canada

**Keywords:** in vivo, ex vivo, metabolomics, proton-only, NMR

## Abstract

Environmental metabolomics provides insight into how anthropogenic activities have an impact on the health of an organism at the molecular level. Within this field, in vivo NMR stands out as a powerful tool for monitoring real-time changes in an organism’s metabolome. Typically, these studies use 2D ^13^C-^1^H experiments on ^13^C-enriched organisms. Daphnia are the most studied species, given their widespread use in toxicity testing. However, with COVID-19 and other geopolitical factors, the cost of isotope enrichment increased ~6–7 fold over the last two years, making ^13^C-enriched cultures difficult to maintain. Thus, it is essential to revisit proton-only in vivo NMR and ask, “Can any metabolic information be obtained from Daphnia using proton-only experiments?”. Two samples are considered here: living and whole reswollen organisms. A range of filters are tested, including relaxation, lipid suppression, multiple-quantum, J-coupling suppression, 2D ^1^H-^1^H experiments, selective experiments, and those exploiting intermolecular single-quantum coherence. While most filters improve the ex vivo spectra, only the most complex filters succeed in vivo. If non-enriched organisms must be used, then, DREAMTIME is recommended for targeted monitoring, while IP-iSQC was the only experiment that allowed non-targeted metabolite identification in vivo. This paper is critically important as it documents not just the experiments that succeed in vivo but also those that fail and demonstrates first-hand the difficulties associated with proton-only in vivo NMR.

## 1. Introduction

The rapid population growth that has occurred over recent decades has resulted in a dramatic increase in the global demand for resources and considerable development in a variety of industrial sectors. This large-scale industrialization is accompanied by a myriad of environmental concerns [1,2], with the impact on water quality at the forefront [3,4]. For example, studies have shown that industrial wastewater, and the stormwater runoff that occurs in heavily industrialized areas, has a significant impact on the health of aquatic systems [5,6]. Both known and novel contaminants released into the environment have been shown to have a negative impact on both human and environmental health [7]. Reviews of toxicological protocols have requested a future paradigm shift to focus on the mechanisms behind toxicity [8]. One way to supply such information is through environmental metabolomics.

Environmental metabolomics is a powerful tool that can be applied to complex biological samples in order to understand the mechanistic responses of an organism that is under the influence of an external stressor [5,9]. One especially promising technique is in vivo nuclear magnetic resonance (NMR) spectroscopy. In addition to being non-invasive, NMR offers the unique ability to obtain information related to the molecular-level changes in an organism in real-time. This method allows for a wide range of metabolites to be observed simultaneously, allowing for investigations into a variety of complex and interconnected pathways [4,10]. This makes it possible to understand why a contaminant is toxic, by providing insight into the mode of action [4,10]. Additionally, in vivo studies render it possible to use the same set of organisms for both controls and exposures, effectively reducing the impact of the variations that naturally occur between individuals [4]. 

To date, a variety of organisms have been employed by metabolomic studies. This list includes earthworms (*Eisenia fetida*) [4,11], freshwater shrimp (*Hyalella azteca*) [11,12], and various species of algae (*Chlamydomonas reinhardtii*, and *Nannochloropsis granulata*) [13,14], fish (*Oncorhynchus mykiss*, *Aeromonas salmonicida*, and *Pimephales promelas*) [15,16,17], and plants (*Lactuca sativa*, and *Solanum lycopersicum*) [18]. The most commonly used model organism for toxicity testing is *Daphnia magna* [19]. *D. magna* are a keystone species within freshwater aquatic systems, with relatively short lifecycles and which are highly sensitive to changes in their environment [19]. Additionally, they are ideal for NMR as they can be maintained inside a standard 5 mm NMR flow system [19]. Typically, in vivo NMR studies are performed with ^13^C-enriched organisms, where ^1^H-^13^C 2D experiments can be used to dramatically improve spectral dispersion. However, following the outbreak of COVID-19 and the Ukraine war (Russia is a key supplier of ^13^C-enriched CO_2_), the costs of isotope enrichment have increased ~6–7 fold. As a result, the culturing of labeled organisms is becoming more and more cost-prohibitive. 

One potential approach to overcome this need for ^13^C-enrichment is to utilize proton-only experiments to obtain metabolic information. However, this is challenging for two key reasons: the overwhelming contribution of lipid signals, and magnetic susceptibility distortions. Lipids are present in high concentrations within an organism due to their role as energy-storage molecules, as well as being a key component of cell membranes and many signaling pathways [20,21]. As a result, lipids dominate in vivo ^1^H NMR spectra, masking nearly all other metabolite signals. 

Magnetic susceptibility distortions [12] often arise when analyzing intact organisms. Different physical structures, for example, cell walls, eyes, stomachs, or exoskeletons, interact with the spectrometer’s external magnetic field differently. In turn, molecules at different locations resonate at slightly different frequencies. When these spin populations are summed across the entire sample, the result is broad peaks, loss of splitting information and additional spectral overlap (compared to an extract) that makes individual metabolite assignment from a standard in vivo ^1^H NMR spectrum impossible. 

To overcome this, the signal broadening must be reduced either by removing the problematic magnetic susceptibility distortions or dramatically improving spectral dispersion [4]. The most effective method of improving spectral dispersion remains 2D ^1^H-^13^C experiments. However, as previously discussed, this is becoming less and less realistic given the rising cost of ^13^C. 

For this reason, it is essential to reconsider the application of proton-only NMR filters and experiments to see what, if any, information can be extracted without ^13^C-enrichment. A wide range of filters are tested here, including relaxation [22,23,24], lipid suppression [11], multiple-quantum [25,26], suppression of J-coupling [27,28,29], 2D ^1^H-^1^H experiments [30,31], selective experiments [32,33], and those exploiting intermolecular single-quantum coherence [12,34]. Two samples are considered, reswollen whole ex vivo organisms and living organisms. Unfortunately, while most filters greatly increase the amount of information that can be obtained from the ex vivo sample, all but the most complex fail on the in vivo sample. Ultimately, this paper is critically important as it documents not just the experiments that succeed in vivo but also those that fail and demonstrates first-hand the difficulties associated with proton-only in vivo NMR. 

## 2. Results and Discussion

### 2.1. Water Suppression

To assess the efficiency of various 1D ^1^H filters and 2D homonuclear (^1^H-^1^H) NMR experiments for NMR-based metabolomics studies, two samples were investigated. The first of these consisted of lyophilized *D. magna* reswollen in a 90:10 solution of D_2_O:H_2_O. The second was an in vivo sample of *D. magna*. For this, an external lock was used, and the live organisms were free to swim in a flow system of 100% water. 

The intact, lyophilized organisms (ex vivo) allow for a much simpler analysis due to the improved line shape and reduced overlap. With the removal of water in the lyophilization process, the hydrogen bonds between the polar lipid heads and the water molecules are broken. This leads to an increase in the packing density of the lipid membranes. In the subsequent swelling process, the compressed lipid structures expand, creating structural imperfections. These physical disruptions in the membrane structures allow the previously trapped metabolites to escape the cell [35]. This effectively reduces the sample inhomogeneity, and, in turn, lessens the impact of the magnetic susceptibility distortions that are typically observed in organisms [12]. 

Reswelling the organisms in a solution primarily composed of D_2_O also gives rise to a less intense water resonance. This effectively reduces the sample inhomogeneity, and, in turn, lessens the impact of the magnetic susceptibility distortions that are typically observed in organisms [12]. Indeed, studying ex vivo organisms has its own merits and previous work has shown that the metabolome is preserved and still maintains signatures resulting from age, pregnancy, etc. [36]. On the flip side, in vivo NMR is the best approach for understanding real-time responses and recovery. As such, these samples are easy to work with compared to in vivo organisms and, therefore, provide a reasonable starting point for the optimization of filter parameters. Once the experimental parameters have been optimized on the ex vivo sample, they can then be applied to the in vivo sample with relative ease. As the in vivo sample is almost entirely composed of water, a more aggressive solvent suppression technique is needed to reduce the water signal. 

The high intensity and broad base of the water signal present in the in vivo sample render the water resonance difficult to suppress. In order to detect components that are present in low concentrations, it is essential to maximize the receiver gain [37,38,39]. A higher receiver gain increases the amplification of the FID allowing the signals of molecules that are present at low concentration and close to the baseline to be discerned. In the presence of large signals, a lower gain is required to avoid saturating the receiver, resulting in decreased sensitivity [40]. As such, water suppression is essential for the analysis of in vivo samples.

**Figure 1:**
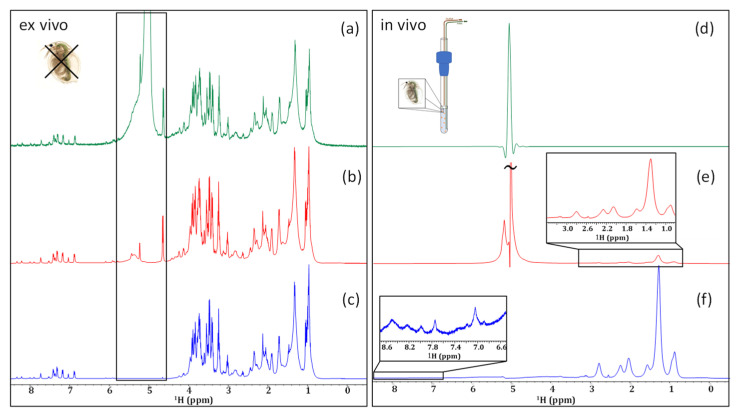
Comparing the application of no water suppression (**a**,**d**), a presaturation (**b**,**e**), and an SPR-W5-WATERGATE (**c**,**f**) in an ex vivo (**left**) and in an in vivo (**right**) sample of *D. magna*.

As seen in Figure 1a, even without the application of any water-suppression techniques, metabolite signals can be observed clearly in the ex vivo sample. Even so, the signals of anomeric protons are superimposed upon the broad base of the water resonance. A simple presaturation experiment, shown in Figure 1b, significantly decreases this water resonance. In this case, the water is suppressed without any major attenuation of the carbo-hydrate anomeric signals at the base of the water.

When analyzing the in vivo sample without water suppression, the receiver could only be set to its minimum value (RG = 1). As a result, no signals from the organisms themselves could be observed (Figure 1d). When presaturation is applied (Figure 1e) a decrease in the intensity of the water signal is observed and, in turn, the receiver gain can be increased (RG = 4.5). The result is that the strongest signals from lipids are visible in the 0.5–to–3.0 ppm range. However, the low receiver gain setting of 4.5 is still only ~2% of the maximum setting possible on a Bruker Avance III NMR system and is far from the optimal dynamic range that is required to detect lower concentration components. 

Previous work has shown that applying a train of selective irradiation pulses before suppressing the water by gradient-tailored excitation with a W5 train (SPR-W5-WATERGATE) is the most effective water-suppression method for natural samples [41]. The use of this more aggressive water-suppression technique on an in vivo sample of *D. magna* (Figure 1f), completely eliminates the residual water. However, it should be noted that the more aggressive technique results in the attenuation of the signals neighboring the water peak. It has been documented that signals within a 1.1 ppm window around the water resonance (0.55 ppm on either side) are subject to some attenuation, and those within 0.4 ppm are suppressed completely [41]. This is clearly seen in the ex vivo spectra where the anomeric signals (see black box) seen with presaturation (Figure 1b) are attenuated with SPR-W5-WATERGATE (Figure 1c).

The application of the shaped W5-WATERGATE sequence is sufficient to suppress the intense water signal present in the in vivo sample (Figure 1f) such that the receiver can be maximized. This allows even the relatively weak peaks in the aromatic region to be detected. Unfortunately, even in the presence of aggressive water-suppression techniques, very limited biochemical information (beyond lipids) can be obtained from the in vivo sample. This is due, in part, to lipids dominating the aliphatic region and masking signals from species with lower concentrations, and, in part, to magnetic susceptibility distortions, as explained in the introduction. This can be seen clearly in Figure 1f. While magic angle spinning (MAS) can be applied to in vivo analyses to reduce the spectral broadening from magnetic susceptibility distortions, it also contributes to a significant stress response in living organisms, limiting its applications for metabolomic studies [42]. As mentioned earlier, another option is to enrich the organisms with ^13^C and acquire 2D NMR, but this is cost-prohibitive and restricts studies to only organisms that can be raised in the lab on a ^13^C diet. Because of these limitations, an investigation into the effectiveness of 1D ^1^H filters and homonuclear ^1^H-^1^H experiments is essential to investigate if useful information can be obtained from organisms without the need for isotopic enrichment. 

### 2.2. Relaxation Filters

Perhaps the most logical filters to implement are relaxation filters as these are used widely in the analysis of complex mixtures, including various metabolomics studies. The two main phenomena utilized by relaxation filters are longitudinal (T_1_) and transverse (T_2_) relaxation [43]. Because large molecules have longer rotational correlation times compared to smaller molecules, they have shorter transverse relaxation times (T_2_) [43]. This gives rise to broader lines, such as those observed for proteins and lipid vesicles. Thus, the application of T_2_ filters can, theoretically, aid in the suppression of these broad signals and reduce spectral overlap. A second approach that is comparable is the application of T_1ρ_ filters. These filters utilize rotating-frame relaxation to reduce or remove signals from macromolecules. One study examined the effectiveness and reproducibility of T_1ρ_ filters compared to a Carr–Purcell–Meiboom–Gill (CPMG) (T_2_) filter in metabolomics studies examining liver and serum samples. It was concluded that the two filters were comparable in their abilities to suppress large molecules, however, T_1ρ_ was found to be more reproducible and better suited for metabolite quantitation [24]. For completeness, both a T_2_ filter and T_1ρ_ filter are examined here. 

Previous work has documented the success of T_2_ filters for the suppression of macromolecules to emphasize the small metabolites present in complex, natural samples [23,44]. Traditionally, the most common method of T_2_ filtering is the CPMG pulse sequence [24,45]. However, the sequence generates echo imperfections, which contribute to decreased signal intensity as the magnetization is not entirely refocused, as well as effects from J-modulation [24,46]. A newer sequence for T_2_ filtering that has been applied in the field of NMR metabolomics is by the periodic refocusing of J evolution by coherence transfer (PROJECT) [11,22]. This sequence utilizes a 90° pulse in the middle of a double-spin echo to form a perfect echo [46]. As a result, J-modulation becomes less problematic in PROJECT, compared to the CPMG sequence, since the echo imperfections are corrected [22]. Metabolomics studies looking at intact plasma [47] and serum [48] have indicated that using the PROJECT variation of the original CPMG sequence resulted in improved recovery of multiplets. Therefore, it is reasonable to expect that a T_2_ PROJECT filter may yield some promising results in terms of lipid suppression, possibly allowing for the identification of individual metabolites when applied to the samples investigated here. 

**Figure 2:**
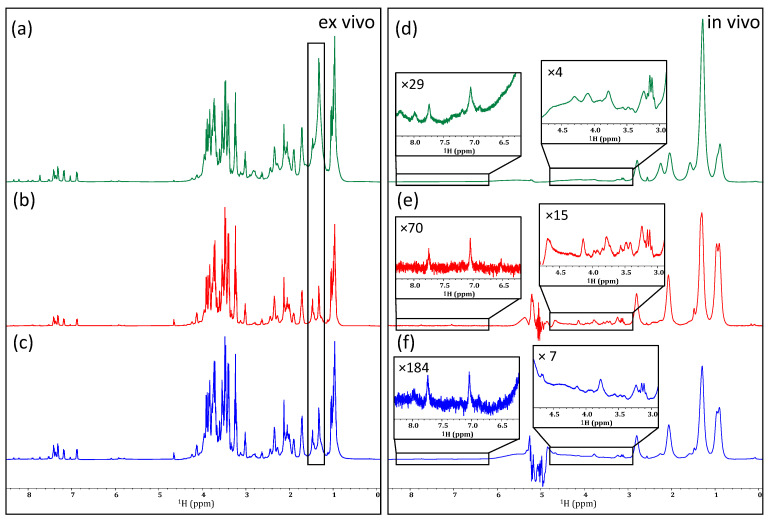
Investigating the application of different relaxation filters to an ex vivo (**left**) and in vivo (**right**) sample of *D. magna*. Comparing an unfiltered spectrum (**a**,**d**) to filtered spectra based on T_2_ relaxation using the PROJECT method (**b**,**e**), and T_1ρ_ relaxation (**c**,**f**). Changes in the intensity of lipid CH_2_ groups are highlighted in the ex vivo sample and vertical expansions are shown for select regions of the in vivo sample.

As demonstrated in Figure 2b, the application of a PROJECT filter to the ex vivo sample of *D. magna* was successful in reducing the intensity of the lipid CH_2_ groups (~1.2–1.6 ppm). An improvement in line shape can be seen as the broad aliphatic signals are reduced. When applied to the in vivo sample (Figure 2e), a reasonable decrease is similarly observed in the lipid signals arising from CH_2_ groups, as well as a moderate decrease in the signal intensity of the neighboring CH_3_ groups. Additionally, with this decrease in signal intensity, a slight improvement in line shape can be observed, especially in the 3.0 to 4.5 ppm region. Past studies have utilized relaxation filters of ~80 ms for the identification of metabolites in complex samples, such as blood, serum, and urine [49,50]. Here it was found that shorter filters had little effect on the lipids and a much more aggressive filter time (400 ms) gave the best discrimination. As seen in Figure 2e, the lipids are significantly attenuated. However, even with the decreased intensity of lipid signals, the aliphatic metabolites remain buried beneath. Similarly, when a T_1_-based filter (T_1ρ_ in this case) [23,24] was employed, a very similar result arose in that the lipids were suppressed in vivo but not to a point where the metabolite signals buried beneath could be recovered (Figure 2f). This is because in vivo there is a continuous distribution of lipids from structural lipids in cell walls/membranes at one extreme, to free intracellular lipids used for energy at the other. While the structural lipids will be preferentially attenuated by a relaxation filter, the free lipids will not. The result is that it is not possible to extend the filter in such a way that the lipid signals are sufficiently suppressed without also attenuating the signals from the metabolites of interest.

### 2.3. Lipid Suppression 

**Figure 3:**
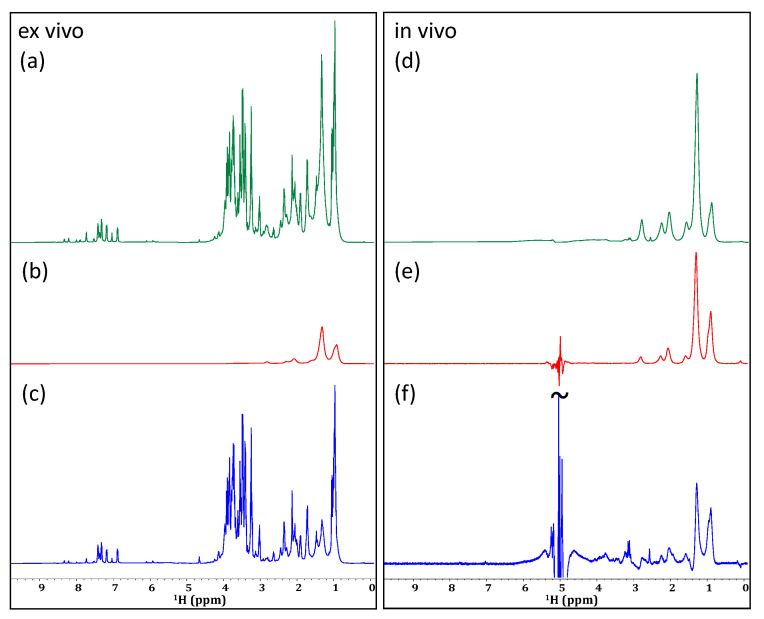
Examining the effectiveness of lipid suppression by subtraction in an ex vivo (**left**) and in an in vivo (**right**) sample of D. magna. A standards SPR-W5-WATERGATE spectrum is shown for each (**a**,**d**), followed by a lipids-only experiment (**b**,**e**), and, finally, the spectrum generated as a result of subtracting a diffusion-edited spectrum from a T_2_-edited spectrum (**c**,**f**).

The relaxation filters discussed above aim to emphasize the small metabolites that are present in complex systems. While only minimal improvement was observed with their application to the in vivo sample, it is reasonable to assume that a technique designed specifically to suppress lipid signals may be more effective. The approach commonly termed “lipid suppression” combines the effect of relaxation and diffusion-editing experiments [11,51]. This method employs a relaxation filter to emphasize the small metabolites present in a sample, followed by a diffusion-editing experiment to suppress the small molecules. The only molecules that pass this diffusion-editing filter are those with long relaxation times, yet restricted diffusion, which in biological samples is mainly aggregated lipids. As such, the resulting spectrum essentially shows only lipids, as seen in Figure 3b,e. Ideally, the difference between these two filters should give rise to a spectrum in which the lipid signals are entirely absent. As seen in Figure 3c, when applied to the ex vivo sample, the resulting spectrum shows a significant decrease in the lipid CH_2_ groups.

When applied in vivo, the difference spectrum (Figure 3f) does show an impressive reduction in the lipid signals. Unfortunately, the lipids are so intense in vivo that the subtraction contains artifacts (twisting) that further complicate the extraction of metabolite information—the result being that the partially twisted residual lipid signals, combined with magnetic susceptibility distortions, still mask the key metabolic information present under the lipids, and the remaining signals are still too broad to permit confident assignment.

### 2.4. Multiple Quantum Filters

Another spectral editing technique that has been used for metabolomics studies, albeit to a lesser extent, uses multiple quantum filters, as these allow for the examination of specific spin-coupled systems. For this, a specific quantum transition is selected based on the number of coupled spins in the system of interest [52]. It should be noted, however, that multiple quantum filters have a couple of limitations. (1) They tend to be relatively insensitive as only a fraction of the total magnetization is selected. For example, when applying a gradient-based double quantum coherence filter, approximately 75% of signal intensity is lost [25] and for higher quantum orders (triple and higher) the losses are far greater. (2) Multiple quantum experiments do not produce absorption mode line shapes and must be processed using absolute-value approaches (i.e., magnitude mode or as a power spectrum) which inherently broaden the peaks. This is far from ideal for complex in vivo samples. 

Typically, multiple quantum filters work in one of two ways: either by gradient selection or by phase-cycling schemes [25]. Phase-cycling approaches generally suffer from poor cancellation of the unwanted magnetization while gradient-based approaches tend to provide cleaner selection [25,26]. As such, the work done here investigates the feasibility of applying gradient-selected multiple quantum filters, rather than those that utilize a phase-cycling scheme. 

**Figure 4:**
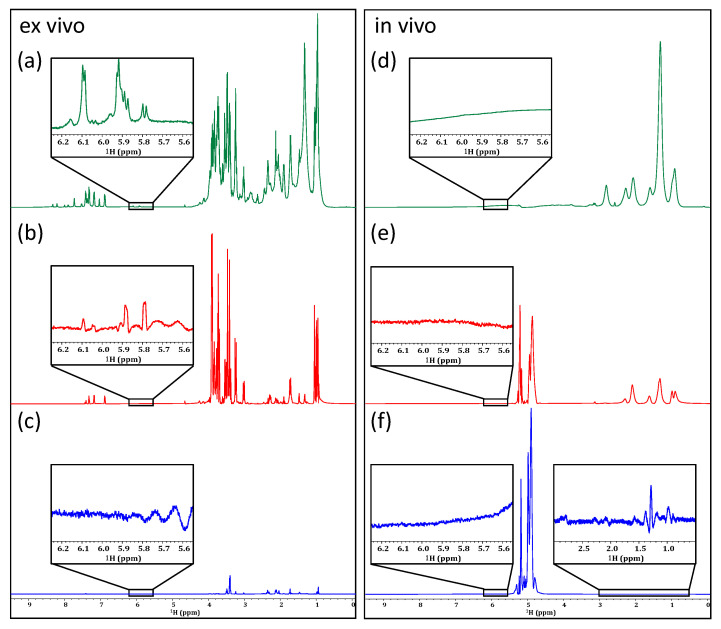
Comparing a standard SPR-W5-WATERGATE spectrum (**a**,**d**) from an ex vivo (**left**) and an in vivo (**right**) sample of *D. magna* to a double quantum gradient selected spectrum (**b**,**e**) and triple quantum gradient selected spectrum (**c**,**f**). Multiple quantum experiments are processed in power spectrum mode; vertical expansions are not.

The application of a double quantum gradient-selected filter to an ex vivo sample of *D. magna* resulted in the effective suppression of lipid resonances as well as the simplification of the spectral profile (see Figure 4b). Processing as an absolute-value power spectrum results in reasonable line shape for the non-phase sensitive data although some artifacts are apparent, especially at the base of the CH_3_ region (~0.9 ppm). The lipids are most likely suppressed due to the relatively long delay (32 ms) required for the evolution of the double quantum coherences. When comparing the non-filtered spectrum (Figure 4a) to the double quantum filtered spectrum (Figure 4b), the filtered spectrum is simplified which could be very useful for the analysis of overlapping resonances. For example, the expansion shown in Figure 4b highlights the simplification of the 5.6 to 6.2 ppm region. This is the chemical-shift range in which energy metabolites, such as adenosine and uridine bases, appear [53]. Signals from ATP itself cannot be observed in the ex vivo sample; however, this is a result of its rapid decomposition following the death of the organism, rather than a failure of the filter itself [54]. Thus, it seems reasonable to presume that when applied to an in vivo sample, this type of filter could permit the monitoring of various energy pathways. Unfortunately, when the double quantum gradient selected filter is applied in the in vivo sample (Figure 4e), none of these energy metabolites can be extracted. This is likely due to a combination of their relatively low abundance, relaxation during the double quantum evolution delay, and magnetic susceptibility distortions. As such, it can be concluded that the double quantum filters are not effective for the tracking of in vivo energy systems, and the persistence of lipid signals in the aliphatic region reduces the potential to extract amino acid side-chain information.

Similarly, the application of a triple quantum spectrum to the ex vivo sample facilitates additional simplification of the spectrum, resulting in a dramatic reduction in spectral overlap (Figure 4c). This, unfortunately, comes with a large loss in the signal-to-noise ratio (SNR). Both Figure 4b,c are scaled to noise such that the signal loss between the double and triple quantum experiments can be gauged. When applied to a living sample, as seen in Figure 4f, very little signal remains, and no additional biochemical information is produced. 

### 2.5. Suppression of J-Coupling

Another method for decreasing overlap in 1D spectra is via the suppression of J-coupling. Arguably, the most state-of-the-art method is pure shift yielded by chirp excitation (PSYCHE). This is a broadband homonuclear decoupling technique that uses a chirp pulse, alongside field gradients, to spatially select sets of spins while suppressing all others [27]. This method has been demonstrated to be very effective in improving spectral dispersion, however, this comes with a considerable loss in sensitivity [27,28]. Some studies have utilized PSYCHE for metabolomics studies on biologically relevant extracts, such as those from soft coral or flowering plants [28,55]. While these studies found that PSYCHE was a reliable and simple method for metabolomics studies of this nature, the same was not true in the case of in vivo *D. magna*. As the approach requires multiple blocks during acquisition, the authors found it impossible to control the water signal, which broke through and distorted the receiver irrespective of the water-suppression method employed. Figure S1 shows an example of an in vivo PSYCHE combined with presaturation; unfortunately, the water swamps the receiver and masks all sample signals. As the approach could not be applied in vivo, its application was not further investigated. 

One other common method for reducing the overlap of 1D ^1^H NMR spectra is through the application of a J-resolved (JRES) experiment. This is a 2D experiment that utilizes a series of spin echoes, along with incremented delays, to encode the horizontal axis with chemical-shift information, and the vertical axis with J-coupling information [27,29]. From this, a tilted horizontal projection can be obtained in which all chemical-shift information is preserved, while multiplets are collapsed into singlets. Additionally, the macromolecules, which have relatively short T_2_ relaxation times, are suppressed. Studies looking at identifying metabolites in plasma, urine, and rat livers, have found that the suppression of broad lipid signals by JRES experiments is more effective than in a typical CPMG filter. These two factors allow for a dramatic reduction of spectral overlap, potentially allowing for the assignment of otherwise hidden or distorted metabolite signals [29,56,57]. However, in some cases, such as a study looking at metabolite identification in follicular fluid, it was found that even with the suppression of lipids, broad signals were still present [57].

**Figure 5:**
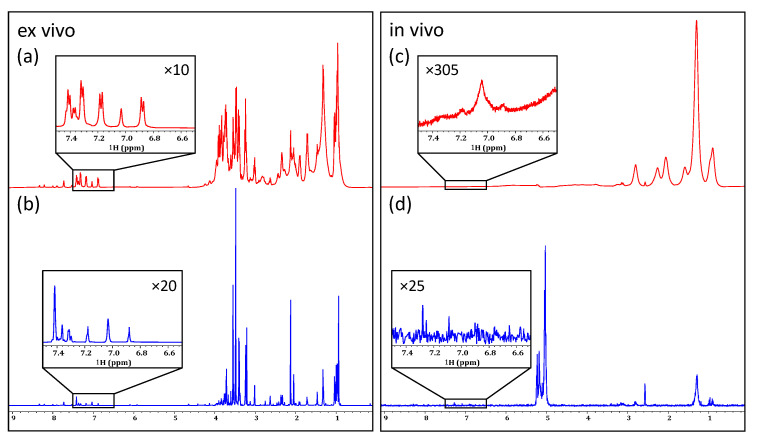
Comparing a standard 1D ^1^H spectrum of ex vivo (**left**) and of in vivo (**right**) samples of *D. magna* (**a**,**c**) to the tilted projection of the JRES experiment (**b**,**d**).

When comparing the unfiltered 1D ^1^H spectrum in Figure 5a with the horizontal projection of a JRES spectrum (Figure 5b), it can be seen that when JRES is applied to an ex vivo sample, the effective removal of multiplicities is achieved as well as an excellent reduction in spectral overlap. This is most evident in the aromatic region, highlighted by the vertical expansions in Figure 5. In contrast, when applied to the in vivo sample in Figure 5d, a dramatic loss of signal is observed. While multiplicities do appear to have been removed, and the lipid signals largely suppressed, most biochemical information is lost. In the current study, the experiment took 2 h and 50 min to acquire at 500 MHz on a prodigy inverse cryoprobe. As such, improving the result at 500 MHz on *D. magna* in a sensible amount of time would likely be very challenging. However, as very weak metabolite signals do appear that are not discernible in the 1D ^1^H NMR alone, the experiment still holds potential for samples with more biomass (i.e., different species, or large diameter cryoprobes that can house more Daphnia) or similar samples at higher magnetic fields. In addition to JRES, various other homonuclear 2D (^1^H-^1^H) NMR experiments have the potential to aid in metabolomic studies.

### 2.6. 2D Homonuclear ^1^H-^1^H Experiments

The application of 2D homonuclear ^1^H-^1^H NMR experiments to complex samples, such as intact organisms, represents an important method for improving spectral dispersion and metabolite identification. One of the most common methods is 2D correlation spectroscopy (COSY). Various types of COSY sequences have been developed, many of which can determine J-coupling constants based on the distance between the peaks of anti-phase doublets [30]. However, the drawback of this is the potential signal cancellation that arises in heavily crowded spectra. Therefore, an alternative method has been developed to allow for the detection of in-phase correlations, using symmetrical excitation. This sequence, known as an in-phase COSY (IP-COSY), results in improved line shape and increased cross-peak intensities [30], making the sequence ideal for in vivo applications [31].

**Figure 6:**
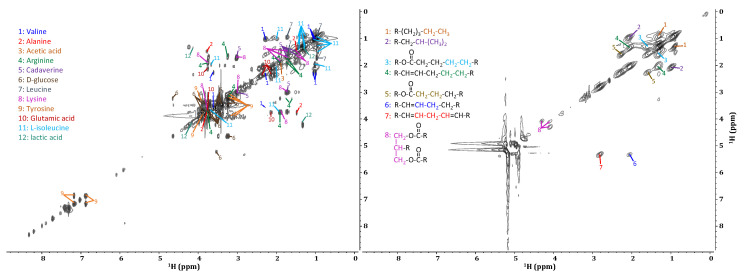
Metabolite identification from the IP-COSY of an ex vivo (**left**) and of an in vivo (**right**) sample of *D. magna*.

As seen in Figure 6, when applied to an ex vivo sample of *D. magna*, the IP-COSY sequence provides detailed information from coupled protons on adjacent carbons. This allows for the identification of a variety of metabolites [53,54]. In comparison, when examining the in vivo spectrum, it is clear that limited information is available. While it is possible to identify the specific lipid components [58,59,60], no other metabolic information can be extracted. This, again, is due to the overwhelming presence of broad lipid correlations. Some studies have achieved reasonably detailed IP-COSY spectra of living organisms from which metabolic information can be obtained. However, this is made possible, in part, by the increased experiment times (6 h 42 min), the application of magic angle spinning (MAS) [42], and the use of shrimp, which have lower total lipid concentrations compared to *D. magna*. Because MAS averages the chemical anisotropy of a sample, reduces the effect of magnetic susceptibility distortions, and narrows the lipid signals, other signals in proximity are easier to discern. However, the longer experiment times necessary to obtain this type of data limit real-time metabolic studies. Additionally, spinning the living samples causes a stress response in the organisms, potentially biasing metabolic processes to the point that true toxicity testing is no longer possible.

An alternative method for reducing the impact of broad lines is to use an adiabatic total correlation spectroscopy (TOCSY) experiment. The use of spin-lock conditions, such as those used in an adiabatic TOCSY, allows for delays to be extended to the point at which they essentially mimic a T_2_ relaxation filter [12,54]. For this reason, an adiabatic TOCSY was attempted on both samples examined here (Figure S2). Similar to the results of the IP-COSY, the ex vivo sample provided detailed metabolic information, whereas the in vivo sample only provided information regarding lipid connectivity. In both the IP-COSY and adiabatic TOCSY, individual metabolite signals could not be identified from the in vivo sample. 

### 2.7. Selective Experiments

**Figure 7:**
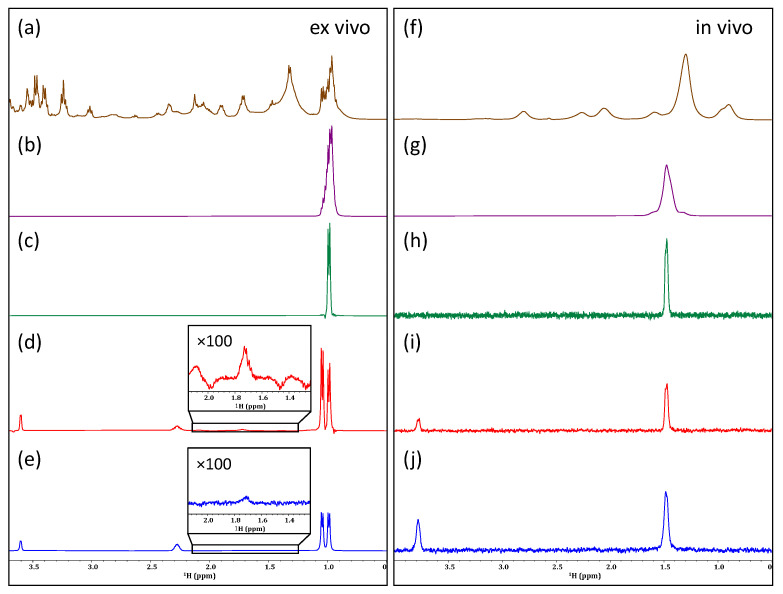
A comparison of various selection experiments for their ability to isolate valine from an ex vivo sample (**left**) and alanine from an in vivo sample (**right**) of *D. magna*. Comparing a basic 1D ^1^H spectrum (**a**,**f**) to a basic selection (**b**,**g**), GEMSTONE (**c**,**h**), GEMSTONE—1D TOCSY (**d**,**i**), and DREAMTIME (**e**,**j**). Vertical expansions showing the relative breakthrough of artifacts the GEMSTONE—1D TOCSY and DREAMTIME spectra recorded on the ex vivo sample.

If only specific metabolites are of interest, one alternative method is to use selective excitation experiments. Building off the basic selection experiment (gradient spin echo) shown in Figure 7b,g, the two leading approaches are GEMSTONE and DREAMTIME. Gradient-enhanced multiplet-selective targeted-observation NMR experiments (GEMSTONE) utilize two adiabatic pulses in addition to a field gradient to encode chemical-shift information. This is conducted following a selective 180° pulse to effectively isolate the multiplet of interest [32]. This sequence allows for the clean selection of a single multiplet, as seen in the case of the ex vivo sample, in which one of the methyl groups from valine was isolated (Figure 7c). In contrast, the basic gradient spin echo (Figure 7b) selects everything in the bandwidth of the pulse and is not selective towards valine. GEMSTONE can then be combined with TOCSY to show all the spins in the system. Figure 7d illustrates the effectiveness of the GEMSTONE—1D TOCSY sequence at isolating all the spins from valine ex vivo.

The second of the two leading approaches is designed refocused excitation and optional mixing for targets in vivo and mixture elucidation (DREAMTIME). The DREAMTIME sequence utilizes a phase-cycled double quantum filter to select J-coupled systems [33,61]. As such, DREAMTIME is highly selective as it requires (1) both spins to be excited exactly at resonances, (2) the spins be adjacent to each other in the molecule, and (3) be coupled over the specified J-coupling [33]. Figure 7e shows the selection of valine from the ex vivo sample. Both GEMSTONE and DREAMTIME work very well, although GEMSTONE has a higher breakthrough of unwanted signals from other compounds (see ×100 expansion), whereas DREAMTIME is much cleaner but slightly more involved to implement. 

Figure 7g–j shows the same experiments in vivo but this time targeting alanine. The standard selection (g) simply selects everything in the bandwidth of the pulse, while GEMSTONE (h) narrows the selection. GEMSTONE—1D TOCSY (i) clearly shows only alanine is selected and the expected α position appears with TOCSY mixing. DREAMTIME (j) is similarly effective also with clean selection of Alanine. Both GEMSTONE—1D TOCSY and DREAMTIME showed remarkable promise in their application to both the ex vivo and in vivo samples. In the in vivo sample, both methods resulted in a similar SNR (~28). The main sensitivity losses with the GEMSTONE sequence are a result of its long delays, which allow for T_2_ relaxation [62], whereas, while DREAMTIME is considerably shorter, its losses in signal intensity can be attributed to the use of a double quantum filter [33]. Based on the results of this study, both GEMSTONE—1D TOCSY and DREAMTIME have great potential for targeted metabolomics studies. However, as GEMSTONE relies solely on a single chemical shift for selection, if two different compounds overlap exactly then it will lead to co-selection. The success of the GEMSTONE experiment with the in vivo sample here was, in part, due to the relatively low overlap in the regions from which alanine is extracted. As such, the highly selective nature of DREAMTIME, as well as its ability to co-select multiple molecules at once [33], mean that it is likely the most versatile method for targeted in vivo studies when more than one molecule is of interest. Nevertheless, while selective methods are a critical tool, non-targeted analysis is irreplaceable when the changes that occur at a biochemical level within an organism are unknown and methods that allow for the simultaneous analysis of the widest possible array of metabolites are essential. 

### 2.8. IP-iSQC

Thus far, all filters discussed here have aimed to reduce spectral overlap to allow for the assignment of metabolites. However, they have not directly addressed the main problem encountered in vivo, namely, magnetic susceptibility broadening. This is most clearly seen in Figure 7i,j, and in both cases alanine is cleanly selected in vivo, however, the signal is still broad given the distribution of slightly different magnetic environments across the organism as a whole. One method to circumnavigate these distortions is to exploit intermolecular single quantum coherence (iSQC) sequences to collect high-resolution NMR spectra in inhomogeneous conditions [12,34]. Specifically, the long-range interactions between solute and solvent molecules are probed over distances greater than the distortions themselves [12]. These long-range interactions can be reintroduced into a liquid state sample by pulse field gradients that break the symmetry and allow strong water-solute dipoles to be observed. When adapted to allow for the acquisition of in-phase data (IP-iSQC), the sequence shows great promise for the analysis of complex, inhomogeneous samples, such as those present in living organisms. As such, the IP-iSQC experiment presented by Fugariu et al. (2017) [12] was explored for its application to in vivo NMR studies. 

As this method works by detecting long-range interactions between protonated solvents and solute molecules, the experiment could not be applied to the ex vivo sample, which was composed primarily of D_2_O. Additionally, since magnetic susceptibility distortions and broad lines do not dominate the ex vivo sample, no additional information can arise from the IP-iSQC experiment. As such, we focus purely on the in vivo case. 

**Figure 8:**
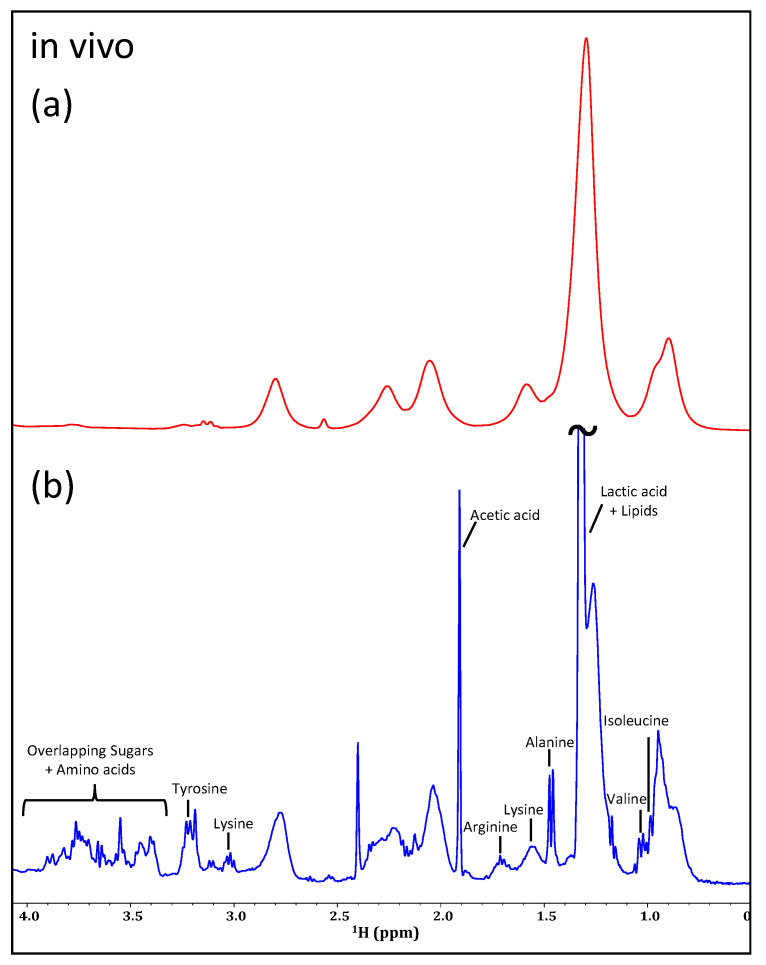
Comparing a standard SPR-W5-WATERGATE spectrum (**a**) of an in vivo sample of *D. magna* to the IP-iSQC spectrum (**b**).

Figure 8 shows the significant improvement in line shape for an IP-iSQC experiment (b) when compared to a standard 1D ^1^H experiment (a). Here, the magnetic susceptibility distortions are suppressed, providing drastically better line shape and spectral dispersion. This allows for the simultaneous identification of various metabolites. The spectrum shown in Figure 8b was recorded with 96 scans in 24 increments, making the total experiment time approximately 3 h. As such, iSQC sequences of this kind show the most promise of the experiments examined here for their application to in vivo NMR metabolomics studies and are, arguably, the only experiment that can provide a non-targeted view of major metabolites in non-enriched in vivo samples. 

## 3. Materials and Methods

### 3.1. Sample Preparation 

*D. magna* were purchased from Ward’s Science Canada in 2010 and have been cultured continuously since then. Cultures are maintained in 4 L glass jars under a 16:8 light:dark cycle. Approximately half of the culture water (dechlorinated and aerated tap water) is replaced twice a week and maintained at approximately 20 °C. The organisms are fed three times a week with *Chlamydomonas reinhardtii* (single-cell green algae), which is grown under the same conditions. To avoid overpopulation, adolescents are separated from adults prior to feeding.

Two samples were prepared for the analysis discussed above. The ex vivo sample was prepared by swelling 40 lyophilized adult *D. magna* in a 90:10 mixture of D_2_O:H_2_O along with approximately 0.01% NaN_3_. The in vivo sample was prepared by transferring 30 adult *D. magna* into a 5 mm NMR tube with a 100% water continuous flow system following the set-up described by Tabatabei Anaraki et al. (2018) [37]. A closed system was used with a Waters Reagent Manager single-piston, pulse-dampened pump along the inlet line and an FMI QV series automated process-control pump along the outlet. An external capillary of approximately 5 μL D_2_O was used for the lock. 

### 3.2. NMR Experiments and Processing 

Spectra were recorded at 278 K using a 500 MHz Bruker Avance III spectrometer and a triple resonance (^1^H, ^13^C, ^15^N) cryogenic prodigy TCI probe. At 287 K (5 °C), the organisms’ metabolisms slow and their long-term survivability increases, as previously reported [42]. Unless otherwise stated, 1D experiments were acquired using a spectral width of 15 ppm, 16k points in the time domain, and a recycle delay of 4 s and processed with a line broadening of 0.3 Hz. The 90° pulse was calibrated for both samples, and a 9.70 μs 90° RF pulse was used for the ex vivo sample, whereas the in vivo sample used one of 9.39 μs.

#### 3.2.1. Water Suppression 

The presaturation and SPR-W5-WATERGATE experiments were recorded with 128 scans and 8 dummy scans. For the presaturation experiment, a 0.0001 W continuous wave irradiation period was used. For the SPR-W5-WATERGATE, a 125 μs binomial delay was used along with a train of 4000 × 1 ms square pulses in the later half of the relaxation delay. 

#### 3.2.2. Relaxation Filters 

Two relaxation filters were tested, both of which also incorporated W5-WATERGATE for the suppression of the water resonance. In both cases, 8 dummy scans and 128 scans were collected on each sample. The first of these was a T_2_ filter following the PROJECT method [22]. For this, 50 loops were used, each of which had 4 separate 2 ms relaxation delays, resulting in a total filter time of 400 ms. The second was a 400 ms T_1ρ_ filter achieved via an X_M16 sequence using a train of 600 µs ca-WURST pulses [63]. 

#### 3.2.3. Lipid Suppression 

For the lipid-suppression filter, previously published methods were followed [11]. Two experiments were acquired and the difference between them was reported. The first of these experiments is a T_1ρ_ filter combined with a bipolar pulse pair longitudinal encode-decode (BPPLED) sequence [64] with the diffusion gradient set to zero power. This spectrum is analogous to a standard T_1ρ_ filter which helps suppress large molecules, such as proteins, leaving mainly metabolite and lipid signals. The second spectrum is collected with the diffusion gradient turned on, such that small molecules with free diffusion are suppressed. Lipids with relatively long T_1ρ_ (due to chain dynamics) survive, producing a spectrum dominated by lipid signals. A weighted subtraction of the lipids-only spectrum from the first spectrum is conducted to give a metabolite spectrum free of lipids. Specific parameters used in this study were a 100 ms T_1ρ_ filter achieved via an X_M16 sequence using a train of 600 µs ca-WURST pulses, a diffusion delay of 100 ms using a 48.15 (or zero for the first spectrum) G/cm diffusion gradient. 128 scans were collected for the spectrum with the diffusion gradient off, and 1024 scans with the gradient on. The additional scans were required given the lower signal in the diffusion-edited spectrum. 

#### 3.2.4. Multiple Quantum Filters

Multiple quantum selection, concatenated with W5-WATERGATE, was performed using gradient coherence selection with 8 dummy scans and 128 scans. For double quantum gradient selection, a 2:1 ratio of gradients was used for the selection of the double quantum coherence and a delay of 32 ms (optimized empirically) to allow double quantum coherence to form. For triple quantum selection a 3:1 ratio and a delay of 40 ms were used. As the multiple quantum experiments were not phase-sensitive, they were processed as power spectra. 

#### 3.2.5. Suppression of J-Coupling 

J-resolved spectra, concatenated with W5-WATERGATE, were recorded using 8k time domain points and 128 F1 increments. A total of 8 dummy scans and 32 scans per increment were collected and processed using a non-shifted sine squared function. The resulting spectra were tilted and symmetrized. RESET_psyches were collected using 4k points, a 30 ms Crp_psyche.10 for psyche element and 200 µs Bip720,50,20.1 for refocusing. Various approaches, including presaturation and W5, were integrated but all failed to control the water in vivo, as such, the approach could not be explored further. 

#### 3.2.6. 2D Homonuclear ^1^H-^1^H Experiments

IP-COSY [30] was concatenated with W5-WATERGATE. A total of 4k time domain points were collected with 128 increments in the F1 dimension. 32 scans per increment were collected using an 11 ms mixing time and a recycle delay of 1 s. The spectra were processed using a sine-squared function that was shifted by π/2 in both dimensions. 

Adiabatic TOCSY was concatenated with W5-WATERGATE. Spectra were acquired in the phase-sensitive mode, using a mixing sequence of 600 µs ca-WURST pulses within an X_M16 mixing scheme and a mixing time of 120 ms. 32 scans were collected for 128 increments in the F1 dimension with a recycle delay of 1 s. The spectra were processed using a sine-squared function that was shifted by π/2 in both dimensions. 

#### 3.2.7. Selective Experiments 

Basic selection was performed using a selective gradient spin echo and 40 ms Gaussian pulse. For the ex vivo sample, valine (1.02 ppm) was selected and in the in vivo sample, alanine (1.48 ppm) was selected. The in vivo experiment utilized 256 scans, whereas the ex vivo experiment needed only 128. Data were processed using an exponential function corresponding to a line broadening of 1 Hz in the transformed spectrum. 

To acquire GEMSTONE spectra, a selective Gaussian shaped pulse of 40 ms was used, flanked by 80 ms crp8,80,20.10 in the presence of a gradient for spatial discrimination. To obtain the in vivo data, 8 dummy scans, 256 scans, and a 1 ms mixing time were used. For the ex vivo spectrum, 8 dummy scans, and 128 scans were used. Data were processed using an exponential function corresponding to a line broadening of 1 Hz in the transformed spectrum. GEMSTONE—1D TOCSY were collected in an identical fashion with a DIPSI spin lock added (120 ms ex vivo, 60 ms in vivo). 

For DREAMTIME all the conditions were the same as GEMSTONE with the exception of instead of one peak, two peaks were targeted per molecule as per the DREAMTIME protocol [33]. Alanine was selected as the target compound using two frequencies (1.48 and 3.78 ppm) and a J-coupling of 7.15 Hz, while valine was selected using 1.02 and 2.27 ppm, and a J-coupling of 9 Hz. 40 ms bi-modulated waveforms were used for selection. 

#### 3.2.8. IP-iSQC 

The IP-iSQC [12] spectrum was acquired with 8k time domain points and a recycle delay of 3.0 s. 24 increments with 96 scans per increment, and an increment delay of 2.0 ms were used. The length of the adiabatic spin lock was optimized empirically on the first increment of the 2D datasets and was set to 100 ms. A gradient strength of −5.4 G/cm was used to allow the solvent–solvent dipoles to manifest. The spectrum was processed with an exponential function corresponding to 1 Hz line broadening and a zero-filling factor of 2. The transformed spectrum was projected along the F2 dimension (direct). The F1 dimension (indirect) was processed using a sine function in combination with a linear backward prediction [10,65] which increased the signal-to-noise while preserving the line shape.

## 4. Conclusions

With the soaring costs of ^13^C-enrichment, alternative options for NMR metabolomic investigations are becoming increasingly sought after. Thus, various proton-only filters and experiments were investigated for their application to in vivo NMR. It has been demonstrated here that aggressive water-suppression techniques, such as SPR-W5-WATERGATE, are necessary to obtain any relevant information in vivo, whereas less aggressive techniques, such as presaturation, can be applied ex vivo to preserve biochemical information in the region surrounding the water peak. More basic filters based on relaxation, diffusion editing, and multiple quantum selection, or 2D homonuclear ^1^H-^1^H experiments were found to provide valuable information in the ex vivo sample. Unfortunately, these filters largely failed for the in vivo sample, although JRES experiments may have the potential to provide additional biochemical information in future applications on organisms with greater biomass. For *D. magna*, the JRES experiment reduced overlap, but the remaining signal was too weak to be of significant benefit. The most potential in vivo comes from the selection experiments and IP-iSQC experiment. DREAMTIME especially holds great promise if metabolites of interest are needed for targeted monitoring. On the other hand, the IP-iSQC was the only experiment that improved line shape and reduced overlap allowing metabolites signals to be assigned in a non-targeted fashion and holds the most promise for proton-only metabolite screening. However, given the reduced spectral dispersion, only ~10 metabolites could be assigned, which is far less than the >60 which can be detected in vivo when ^13^C-enrichment is used [54]. As such, despite its cost, ^13^C-enrichment is still recommended for comprehensive metabolite assignment and monitoring in vivo where feasible. 

## Data Availability

The data presented in this study are available on request from the corresponding author.

## Supplementary Materials

The following supporting information can be downloaded at: https://www.mdpi.com/article/10.3390/molecules28124863/s1. Includes, figures showing ^1^H PSYCHE in vivo (Figure S1. (a) Standard 1D ^1^H in vivo spectrum. (b) Attempt at applying ^1^H PSYCHE to an in vivo sample. Unfortunately, due to the excessive water signal it was not possible to extract any information in vivo using PSYCHE) as well as adiabatic TOCSY ex vivo and in vivo (Figure S2. Metabolite identification from the adiabatic TOCSY of an ex vivo (left) and in vivo (right) sample of *D. magna*. Only lipids are detected in vivo and the structural units giving rise to signals are labelled).
