# Supplementary material for: Exploring Proton-Only NMR Experiments and Filters for Daphnia In Vivo: Potential and Limitations
Source: Molecules. 2023 Jun 20;28(12):4863. doi: 10.3390/molecules28124863 (PMC10301599; doi:10.3390/molecules28124863)
Supplement: Supplementary file 1 [file molecules-28-04863-s001.zip › molecules-2455430-supplementary.pdf]

Supporting information

Table of Contents

S1. In vivo PSYCHE S2

S2. Adiabatic TOCSY S2

S1. In vivo PSYCHE

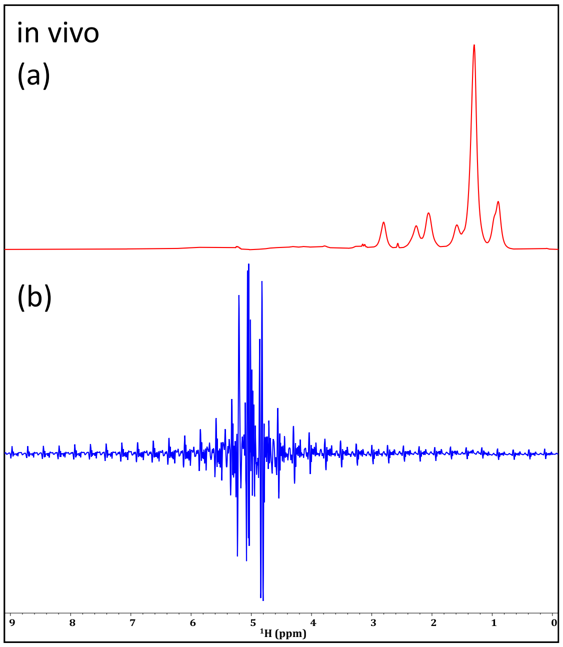

Figure S1. (**a**) Standard 1D ^{1}H in vivo spectrum. (**b**) Attempt at applying ^{1}H PSYCHE to an in vivo sample. Unfortunately, due to the excessive water signal it was not possible to extract any information in vivo using PSYCHE.

S2. Adiabatic TOCSY

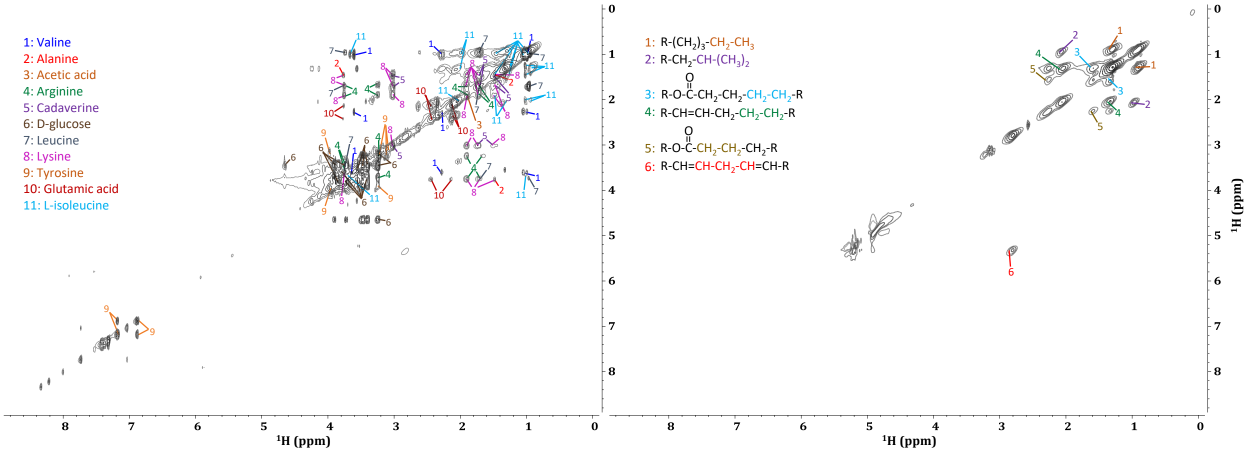

Figure S2. Metabolite identification from the adiabatic TOCSY of an ex vivo (**left**) and in vivo (**right**) sample of *D. magna*. Only lipids are detected in vivo and the structural units giving rise to signals are labelled.
